# Combination of the Check-All-That-Apply (CATA) Method and Just-About-Right (JAR) Scale to Evaluate Korean Traditional Rice Wine (*Yakju*)

**DOI:** 10.3390/foods10081895

**Published:** 2021-08-16

**Authors:** Sanghyeok Lee, Han Sub Kwak, Sang Sook Kim, Youngseung Lee

**Affiliations:** 1 Department of Food Science and Nutrition, Dankook University, Cheonan-si 31166, Korea; leesh940324@gmail.com; 2 Research Group of Food Processing, Korea Food Research Institute, Wanju-gun 55365, Korea; hskwak@kfri.re.kr (H.S.K.); sskim@kfri.re.kr (S.S.K.); 3 Korea Food Research Institute, KFRI School, University of Science and Technology, Wanju-gun 55365, Korea

**Keywords:** CATA, RATA, JAR, penalty analysis, yakju

## Abstract

This study aimed to compare a variant of the check-all-that-apply (CATA) method, CATA with just-about-right (JAR) scales (CATA-JAR), with the CATA and rate-all-that-apply (RATA) methods for evaluating 12 Korean traditional rice wines (*yakju*). All consumers (*n* = 312) assessed each sample on a 9-point hedonic scale and were asked to fill out the CATA, RATA, or CATA-JAR questionnaire using a 5-point JAR scale. The frequency and percentage of terms with significant differences among CATA-JAR samples were significantly higher than those for the CATA method. The regression vector (RV) between the sample and term configurations of the three methods were all over 0.84, indicating that all methods were similar in terms of product and term usage. Regarding the stability of the sample configurations, CATA-JAR could derive a stable value with the lowest number of consumers (*n* = 25). For the CATA-JAR method, significant penalties for each attribute and product were successfully calculated using the t-test and bootstrapping technique, to identify any attribute detrimental to liking for each product. Overall, considering its better performance in discriminating products and stability, the CATA-JAR method may be used when comparing samples with subtle differences in attributes.

## 1. Introduction

*Yakju*, a Korean traditional rice wine, is often made of cooked rice, *nuruk* (starter based on grain fermentation), and yeast [1,2,3]. Similar to other traditional Korean rice wine (i.e., *makgeolli*), *yakju* is typically produced using yeast through a parallel process of saccharification and alcohol fermentation. This process requires additional water for the mixture of *nuruk* and yeast [4,5]. Rice is first washed, soaked, and steamed; then, the steamed rice is cooled to room temperature (25 ℃) and then mixed with *nuruk* and yeast.

Rapid sensory profiling methods based on consumer perceptions have been actively explored in sensory science because conventional descriptive analysis requires excessive time and effort to produce reliable results [6]. The rapid sensory profiling methods widely used include projective mapping [7], polarized sensory positioning [8], free multiple sorting [9], napping [10], free-choice profiling [6], flash profile [11], pivot profile [12], and check-all-that-apply (CATA) methods [13]. Among these, the CATA method has gained considerable popularity than other methods because it has the advantages of being a simple and versatile technique for consumer perception, and being easy to implement by both trained or untrained consumers [14,15,16,17]. This methodology has been reported to be robust in providing reliable information about sensory characterization and can discriminate samples sufficiently [18]. However, it cannot fully replace descriptive analysis that can identify and quantify sensory attributes well defined by highly trained panelists [19]. Studies on the CATA method have been extensively used to gain consumer perception of various foods such as cooked rice [13], wines [15], yogurts [20], orange juices [21], beer, tea, strawberries [22], vanilla ice creams [23], wholegrain bread [24], fish [25], apple purees [26], and sparkling wines [27].

However, one limitation of the CATA method is that it does not reflect or measure the intensity of perceived sensory attributes [28]. As the binary responses elicited by the CATA cannot directly measure the intensity of sensory attributes evaluated, it is impossible to directly quantify the intensity of sensory attributes, making it difficult to compare products with similar sensory profiles [29]. Various efforts have been taken to overcome these shortcomings of the CATA method, including the introduction of CATA variants. One representative is the rate-all-that-apply (RATA) method [30,31].

The RATA method combines the CATA questions with intensity scales [31]. The intensity scales applied in the RATA questions can be used in various formats, including 3, 5, or 15 points [28,32]. Unlike the CATA method, the RATA method is designed to quantify the intensity of attributes, especially for highly similar products. In addition, similar to the CATA method, it is possible to implement it with untrained consumers, providing a better discrimination between a product’s sensory profiles [33]. Practical applications of the RATA method have been validated for various foods, including wine [34], milk powder [35], black tea [36], and apples [31]. Ares et al. [29] reported that the RATA method had superior sample discrimination and configuration stability compared to those of the CATA method.

Ares et al. [37] indicated that the RATA method was not similar to the descriptive analysis in terms of quantifying the sensory profiles of products, showing no high correlation between the intensity of attributes of the two methods. Ares et al. [38] also reported that consumers not trained using intensity scales often lacked a consensus in their evaluations and showed incongruence between the RATA method and descriptive analysis. The authors reported that only those attributes well known or that had distinct differences between samples (sweet, sour, salty, etc.) showed good correlations between the RATA method and descriptive analysis. In contrast, when using the RATA method for attributes that are unaccustomed or difficult to define (fibrous, ammonia odor, salami scent, etc.), a lower discrimination was observed between products when compared with the descriptive analysis [38]. Oppermann et al. [33] reported that it would be possible to reduce these limitations by training consumers on the definition of attributes and how to use the scale to improve the familiarity and cognition of products. However, additional training for the consumers to overcome these limitations could offset the time and cost effectiveness of using the RATA method [37].

The JAR scale has been widely used in consumer research with liking or sensory scales [39], although the hedonic perception of products can be biased due to consumers’ analytical attention to their liking cognition [40,41,42], which has been controversial until now. The JAR scale quantifies the optimum intensity of sensory attributes using bipolar scales (i.e., 1 = “too weak,” 3 = “JAR,” 5 = “too strong”) [43]. The concept of the JAR scale is based on an ideal point model. The level of JAR is considered as the standard, and if the level deviates from the JAR, it implies a deviation from the ideal point (Rothman and Parker, 2009).

Ares et al. [44] compared the 5-point JAR scales to CATA questions with intensity and hedonic connotations, similar to the anchors on the JAR scale, for six consumer studies. The JAR anchors were included in the CATA questions. Consumers evaluated the CATA method and then finished a series of JAR scales by comparing the two methodologies. It was observed from their study that both methodologies showed similar insights about the most pertinent deviations from the ideal, despite differences between the two methodologies. They also compared the performance of penalty analysis (PA) between CATA questions and JAR scales, indicating that deviations from the ideal were similarly identified by these two approaches.

Ares et al. [29] observed a substantial heterogeneity among consumers who used the intensity or applicability scales combined with CATA questions due to different cognitive processes in attribute applicability and intensity in evaluation. As described by Ares et al. [38], sensory characterization with consumers using intensity scales is not recommended, because the consensus for sensory attributes and the ability to discriminate samples was low. Here, a JAR scale would be an interesting alternative to the rating scale, and it would be interesting to examine how the cognitive processes of using a JAR scale differ from those on the intensity scale.

To the best of our knowledge, there is no study comparing the performance of the combination of the JAR scale and CATA questions with the CATA and RATA methods. An additional function of using the combined use of a JAR scale with CATA questions is to use PA. PA determines the effect of specific attributes that deviate from the optimal (i.e., levels above and below the JAR level) on the hedonic level of a product [45]. The current study aimed to compare the performance of a CATA method with a JAR scale (referred to as CATA-JAR hereafter) as a rapid sensory profiling method to the CATA and RATA methods for evaluating Korean traditional rice wine (*yakju*).

## 2. Materials and Methods

### 2.1. Samples

Twelve retail *yakjus* were selected from a marketplace in Korea. As *yakju* has supplementary ingredients besides rice, and *nuruk* has gained consistent popularity because of its unique flavors and functionalities, all 12 samples used in this study contained additional ingredients with a broad range of sensory properties (Table 1). The expiration dates of samples were all identical (i.e., 12 months) and the samples were immediately kept at 4 °C before analysis.

### 2.2. Consumer Test

This study was approved by the Institutional Review Board of Dankook University in Korea (approval number: DKU 2019-04-021-001). A total of 314 participants (155 males and 159 females) were recruited in and around Dankook University through advertisements via flyers and social networks. Qualification criteria included adults over 20, no allergy to alcohol, possibility of consuming alcohol, and *yakju* consumption at least once a month. As this study was focused on the methodological approach, which aimed at introducing and validating a CATA-JAR method, such a demographic and behavioral data analysis of consumers was not included in this study. Consumers were randomly assigned to one of the three experimental methods: one group of 100 consumers (50 males and 50 females) for the CATA method, another group of 107 consumers (52 males and 55 females) for the RATA method, and a group of 107 consumers (53 males and 54 females) for the CATA-JAR method. A between-subjects design was used to compare the three methods.

A total of 35 mL of each sample was served in a paper cup (70 mL) at 18 ± 1 °C with three-digit random codes. Consumers were instructed to rinse their mouths with filtered water between the evaluations. Consumers were asked to put the entire sample in their mouth, swirl it across their tongue, and expectorate it because of intensive sensory fatigue from alcohol [46]. The consumers evaluated the samples in two sessions and provided six samples per evaluation session. The samples were served in sequential order following the Williams Latin Square design. The order of attributes was identical within consumers for sessions, while randomized between consumers. All consumers took a 20 min break between sessions in consideration of sensory fatigue. All groups of consumers rated the overall liking (OL) of each *yakju* on a 9-point hedonic scale (“1 = extremely dislike,” “5 = neither dislike nor like,” and “9 = extremely like.”) and were asked to fill out the CATA, RATA (“1 = very weak,” “3 = medium,” “5 = very strong”) using 5-point intensity scales, or CATA-JAR (“1 = too little,” “3 = just about right,” “5 = too much”) questions using 5-point JAR scales. Each questionnaire comprised 27 attributes (2 appearances, 11 aromatics, and 14 flavors). The OL data were collected before the CATA, RATA, or CATA-JAR questions.

### 2.3. Data Analysis

An analysis of variance (treating the sample as a fixed effect and consumer as a random effect) was performed to find significant differences between CATA, RATA, and CATA-JAR methods regarding the OL values, using a Tukey’s test with α = 0.05. Correspondence analysis (CA) based on the chi-square distance with confidence ellipses was obtained on the matrix with the frequency of each term for each sample. Confidence ellipses around the samples were obtained using bootstrapping.

Agglomerative hierarchical clustering (AHC) analysis, considering Euclidean distances and Ward’s aggregation criterion, was performed to assess how the samples with similar sensory characteristics were grouped for each method. AHC was performed on sample coordinates in the first and second dimensions using the three methods. The regression vector (RV) coefficient was obtained using the first and second sample coordinates to examine the similarity of the sample configurations from the three methods. Significant differences for each of the sensory attributes for each method were determined using Cochran’s Q test, whereas differences in the total number of attributes elicited for consumers to describe samples were compared using Fisher’s exact test.

The number of consumers required to obtain a stable configuration for the sample and attribute from the three methods was estimated using a bootstrapping resampling technique. According to Blancher et al. [47], the stability of the sample and attribute configurations is established if the simulated configurations show similar results to the original configuration of samples and attributes. A random resampling process comprising 1000 subsets of consumers (equal to the total number of consumers used in this study) was performed using the bootstrapping technique. The similarity between sample configurations from each subset and the original sample configuration was compared using the RV coefficient [48]. Average coefficients and standard deviations were calculated for each consumer.

The PA was applied to the CATA-JAR results for all 27 attributes on the JAR scale. Categories on each side of the JAR level (i.e., “too little” = TL, and “too much” = TM) collapsed, whereas the percentage of consumers and their corresponding average OL scores for the categories TL, JAR, and TM were calculated. As a next step, penalties were calculated by subtracting the average OL scores for the JAR group from the average OL scores of the TL or TM groups. Significant penalties for each attribute and product were determined using t-tests. We also performed a bootstrapping technique where the t-test might not be satisfactory, because of the small number of responses. The bootstrap estimate of variability was calculated through 10,000 sets of resampling of the data pairs (i.e., OL and JAR scores).

Notably, any attribute with a missing check in CATA-JAR must be appropriately treated for proper PA analysis. We employed the concept of the best estimation threshold, which implied that an individual’s threshold is the concentration they can detect 50% of the time [49]. Thus, only attributes checked by over 50% of the total consumers were used for the PA analysis. All statistical analyses were performed with XLStat 2016 (Addinsoft, Paris, France) and the R language using FactoMineR.

## 3. Results and Discussion

### 3.1. Consumer Acceptability of Different Methods

Table 2 presents the mean OL values obtained from the consumers for each of the methods. No significant difference was observed in the OL values among the three methods, thus suggesting that the ranking of OL in products was similar for all methods. This indicated that the effects of different methods were minimal on OL when OL questions were concurrently used with CATA, RATA, and CATA-JAR methods. This finding contradicts studies [40,41,42] that reported that using JAR scales with hedonic scales changed the hedonic perception of the sample because the JAR scales could force consumers to focus their attention on specific sensory attributes. However, the results were in line with the studies showing that the CATA method did not affect consumer acceptability and did not induce hedonic bias [22,50,51,52]. This implied that the different methods applied following OL scales are unlikely to affect the hedonic scores, and thus consumers’ perception of the sensory characteristics among the methods could be directly compared without inducing hedonic bias.

### 3.2. Term Usage and Sample Differences for each Method

The frequencies of using sensory terms for each method are shown in Table 3. Consumers used significantly larger percentages of sensory attributes to describe samples when using the RATA (29.4%) or CATA-JAR (28.7%) compared with the CATA (26.6%) method. This is consistent with previous studies reporting that the frequency of term usage was higher for CATA combined with the intensity scale than for the simple CATA method [31,53].

Vidal et al. [31] reported that consumers perceived attributes more analytically when performing CATA combined with intensity scales than when using the CATA method; the satisfying response strategies [54] seemed less pronounced for the RATA and CATA-JAR methods in this study. This is because consumers needed more analytical and cognitive efforts to answer RATA or CATA-JAR questions than CATA questions. For the CATA method, the satisfying response strategies allow consumers to not select all attributes, especially those with a low intensity, to describe the product, but instead only select the most salient attributes to characterize the product [31]. In contrast, for the RATA or CATA-JAR methods, consumers would tend to find more detailed attributes of the products on their analytical mindset, possibly selecting a greater number of attributes. Another possible reason is that consumers’ cognitive processes for CATA and CATA-JAR may change due to an additional rating task [31]. Identifying an attribute with a low intensity could make consumers focus on selecting more terms compared to when they need to identify attributes to describe products (i.e., CATA).

The distribution of the RATA and CATA-JAR scores is shown in Table 3b,c. The middle points of the scales (3: “medium” and “JAR” for RATA and CATA-JAR, respectively) were the most frequently used for both methods. In addition, “low” scale anchors were more frequently used than “higher” anchors. This is consistent with a previous study demonstrating that consumers who used the RATA method most frequently used the middle point of the intensity scale, followed by the “low” intensity anchor [31]. This could be attributed to the effect of central tendency, which has been well established in sensory science [55]. Vidal et al. [56] reported that although the attribute is identified, some consumers tend not to select the attribute or rate it a lower score to avoid extreme anchors in the RATA method. Although the unique characteristics of scales in the RATA and CATA-JAR methods were different, the distribution of anchors of the two methods was similar, indicating that consumers avoided extreme anchors for both methods.

However, it is interesting to note that the middle point was selected more for CATA-JAR (10%) than for RATA (7.8%). Different cognitive processes could be present when evaluating intensity and JAR scales, especially for the middle points of the scales (“medium” and “JAR” for RATA and CATA-JAR, respectively), allowing consumers to consider sensory attribute tasks differently. Specifically, consumers may use different cognitive processes when evaluating an attribute at the center of an intensity scale and JAR scale. For example, the sweetness intensity of a food may be perceived as “not enough” by some, but “too sweet” or “just about right” by others. A regular consumer and another one on a diet may rate the intensity of sweetness of the food the same, but the latter would always rate the food as “too sweet.” Thus, intensity scales can be considered an objective quality indicator, whereas JAR scales are the combined measurement of attribute intensity and consumer acceptability as a subjective quality indicator [39]. Lawless [57] supported the idea that dissimilar cognitive processes may be engaged with the attribute evaluation for the applicability and intensity. Therefore, Ares et al. [37] suggested using attribute intensity scales for simple products and applicability scales for sensory characterizations of complex products (i.e., wine).

Cochran’s Q test (Table 3d) was conducted to determine significant differences among samples for each sensory attribute [29]. The percentages of significant attributes among samples were as follows: CATA (92.6%), RATA (100%), and CATA-JAR (100%). This indicated that both the RATA and CATA-JAR methods showed a higher product discriminability than CATA while showing comparable performances regarding similarities and differences among samples for most sensory attributes. This result is consistent with that of Ares et al. [29], who reported that the percentage of attributes with significant differences among samples of the RATA method was equal to or higher than the CATA method. This could be because consumers were more likely to generate an analytical mindset focusing on attribute intensity or JAR [58].

### 3.3. Correspondence Analysis (CA) with Confidence Ellipse of Samples and Agglomerative Hierarchical Cluster (AHC) Analysis

The percentage of variance explained by the first and second dimensions of CA was higher than 70% for all the three methods (Table 3e). Higgs [59] reported that it would be adequate for market research needs if at least 70% of the variation is explained by two dimensions.

As shown in Table 3f,k, the RV coefficients between the sample and attribute configurations of all the three methods were 0.90 or greater, indicating that all methods were very similar. According to previous studies by Ares et al. [29] and Vidal et al. [31], who compared CATA and RATA methods for various foods (i.e., sliced bread, milk desserts, apples, gummy lollies, yogurt labels, peanuts, tinned pineapple, raspberry coulis, and fruitcake powdered drinks), a high similarity was observed between the two methods. Jaeger et al. [51] also reported a high similarity between the CATA method and concurrent use of JAR and CATA methods for various foods (i.e., fruit cake, mussels, milk chocolates, pear, apple, peanuts, and green kiwifruit). CATA-JAR and RATA had the highest RV coefficients of the sample (0.978) and term (0.958) configurations in this study, which was the most similar among the three methods. This again suggested that these two methods were characterized by sensory attributes similarly and showed highly similar product configurations.

**Figure 1:**
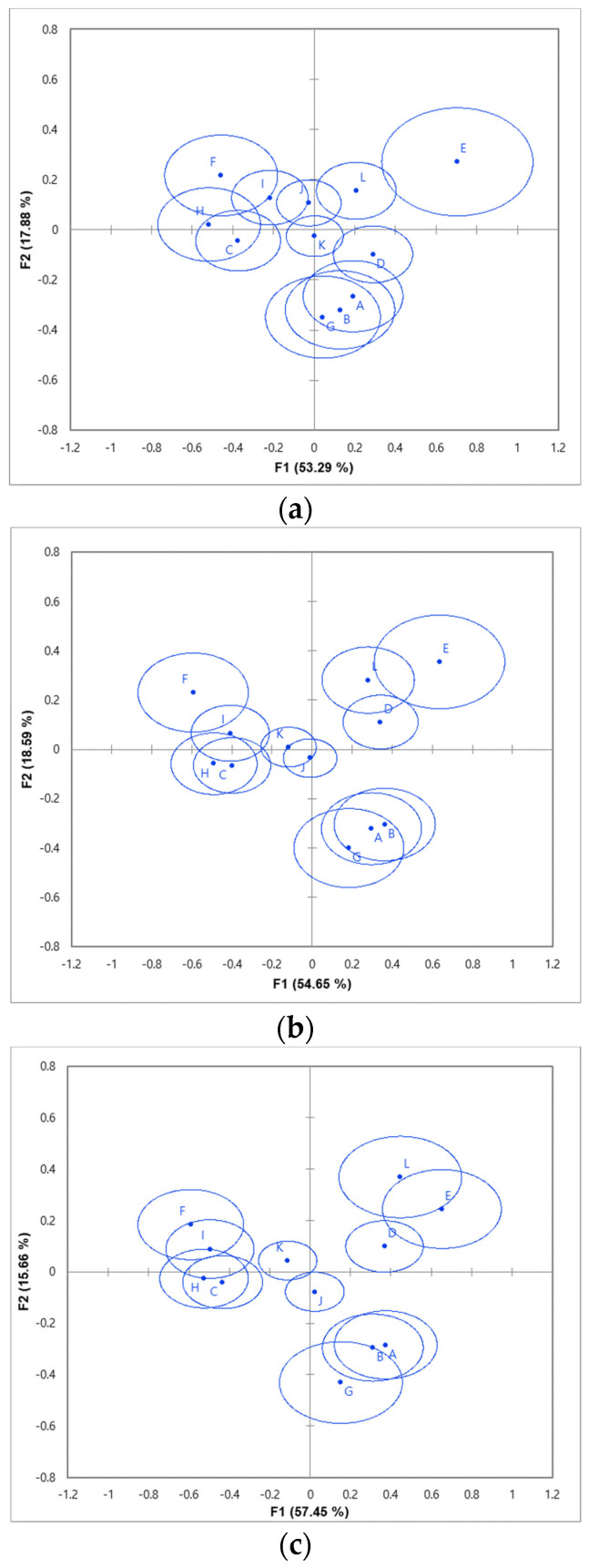
Representation of the *yakju* samples in the first two dimensions of correspondence analysis data from (**a**) CATA method, (**b**) RATA method, and (**c**) CATA-JAR method. Product codes are as in Table 1.

The confidence ellipse of the products on the CA map for each method is shown in Figure 1. Husson et al. [60] reported that the confidence ellipse in CA represents the significance of the difference between the products. For the CATA method, the confidence ellipses of all products seemed to overlap with one group. Products with the confidence ellipse from the RATA method (Figure 1b) were divided into three groups, whereas products with confidence ellipses were divided into four groups for CATA-JAR (Figure 1c). Based on the study by Vidal et al. [56], the RATA and CATA-JAR methods had a higher sample discrimination than the CATA method did because these methods showed a larger number of groups with confidence ellipses.

**Figure 2:**
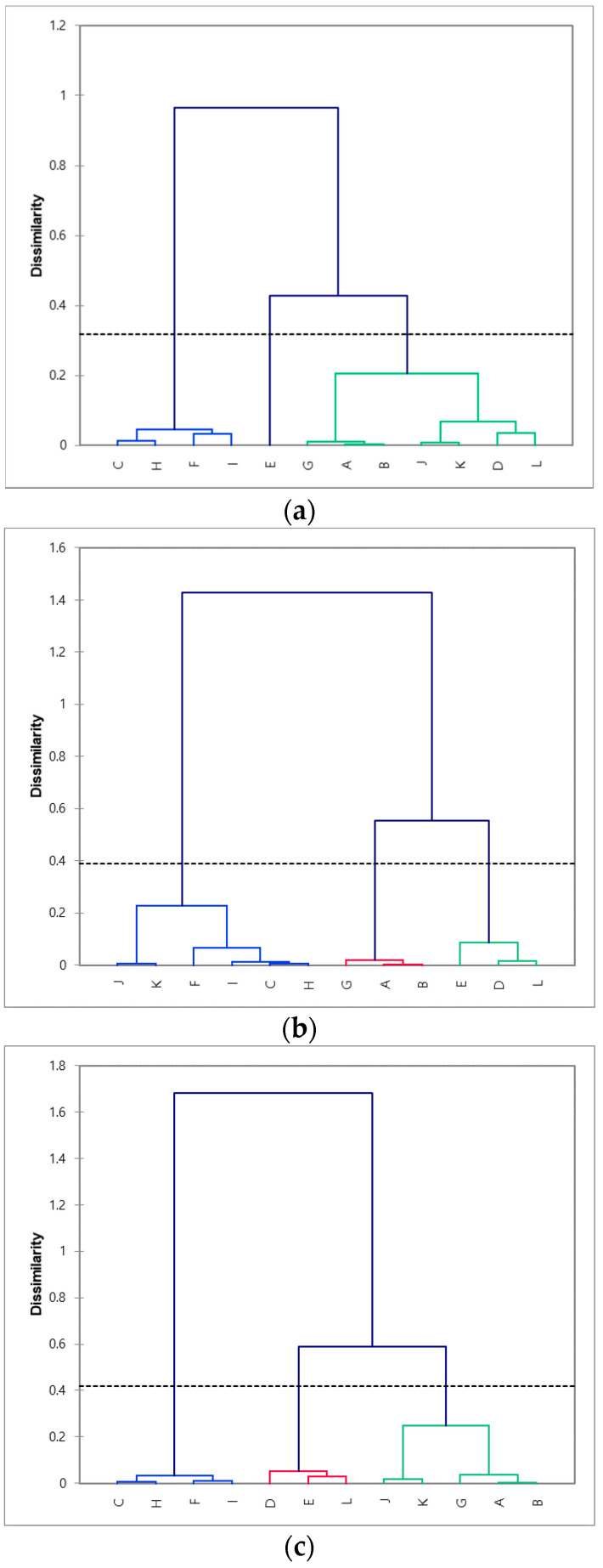
Dendrograms from agglomerative hierarchical cluster analysis performed on *yakju* samples for (**a**) CATA method, (**b**) RATA method, and (**c**) CATA-JAR method. Product codes as in Table 1.

Cluster analysis is a useful multivariate technique for segmenting objects into homogeneous groups based on the differences in individual characteristics [45]. Samples were classified by AHC analysis, based on the coordinates of the samples, in the first and second configurations of the dimensional maps from the three methodologies (Figure 2). The configurational similarity was assessed using the dendrograms generated through AHC analysis [61]. For the CATA method, three groups were identified: the first group included samples C, F, H, and I; the second group included samples A, B, D, G, J, K, and L; and the third group included only sample E. The following three groups were identified for the RATA method: the first group (samples D, E, and L), second group (samples A, B, and G), and third group (samples C, F, H, I, K, and L). The same three groups were also generated for the CATA-JAR. The largest cluster contained samples C, F, H, I, J, and K, and the remaining two clusters contained samples D, E, and L and A, B, and G, respectively. The HCA confirmed the considerable configurational similarity between RATA and CATA-JAR. All the samples were in the same clusters, except for samples J and K. The CATA method seemed to be the least congruent among the three methods because sample E was separated from the other samples.

### 3.4. Stability of Sample Configurations

**Figure 3:**
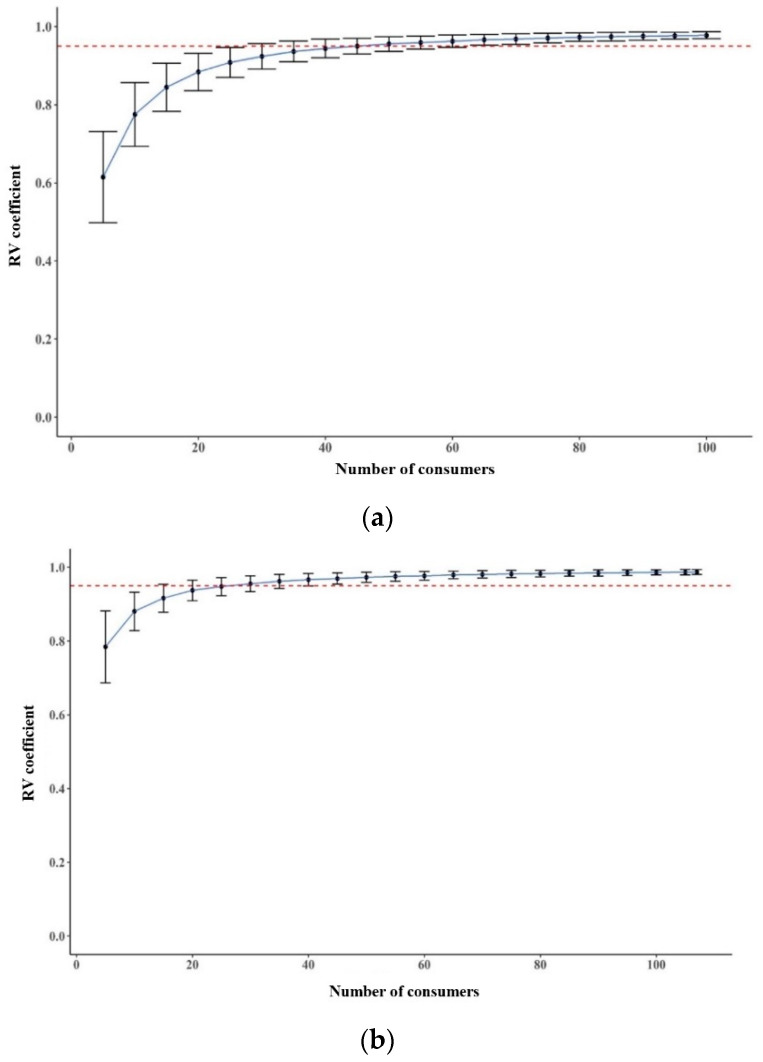

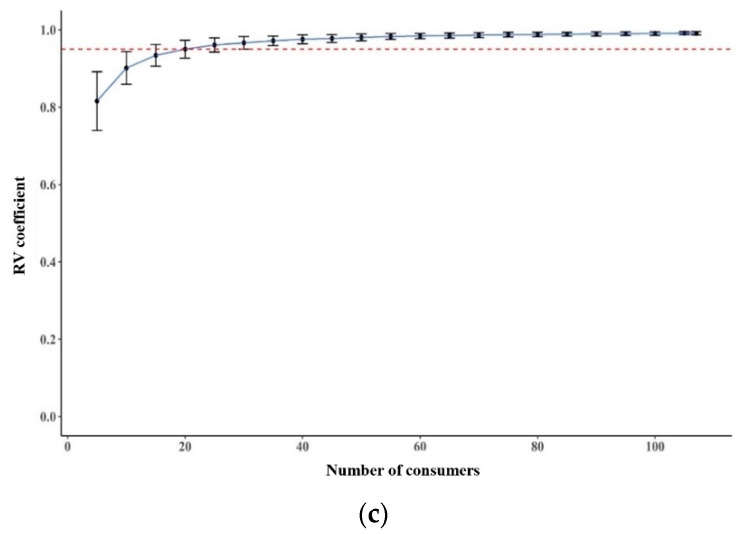
Average regression vector (RV) coefficient of samples in terms of original configuration of samples as a function of the number of consumers considered in the resampled simulated panels for each method. Vertical bars correspond to standard deviations: (**a**) CATA method; (**b**) RATA method; (**c**) CATA-JAR method.

The stability of the sample configurations for each method was compared using a bootstrapping technique [62]. As shown in Figure 3, the average RV coefficient of sample configurations increased for all methods as the number of consumers in the simulated dataset approached the original number of consumers used in the current test, whereas its standard deviations decreased. An average RV coefficient of 0.95 has been indicated as a stable sample configuration compared with the original sample configuration [46]. Considering this criterion, the RATA and CATA-JAR showed more stable sample configurations than the CATA method did, regardless of the number of consumers in the simulated panel. The number of consumers required to reach stable sample configurations (RV coefficient of ≥0.95) was approximately 50 for CATA and 30 and 25 for RATA and CATA-JAR methods, respectively. This suggested that inducing consumers to be analytical by rating attributes can help stabilize sensory configurations. This is consistent with a previous study in which the RATA method provided more stable sample configurations than the CATA method did for milk desserts, bread, lollies, etc. [29]. Ares et al. [63] reported that sample configurations met a stable criterion (RV coefficient ≥0.95) in at least 8 to 90 consumers in the CATA method where sample differences were large, whereas when sample differences were small, an RV coefficient equal to 0.95 was not acquired.

### 3.5. Penalty Analysis (PA) of CATA-JAR Method

PA is a useful tool to analyze the potential penalty paid by the product regarding reduced OL for not being “just about right” on an attribute, and the penalty is often called the mean drop in OL [64]. PA with data from CATA questions was introduced based on the ideal profile concept [24,29]. Ares et al. [29] identified drivers of liking determined by the degree of reduction in OL due to deviations in sensory attributes between observed and ideal products through CATA questions. However, this approach differed from the one used in this study because the JAR scales were directly combined with the CATA question, and PA analysis was performed on the CATA attributes.

Table 4 shows the results of the PA obtained using the CATA-JAR method. Products and attributes given significant penalties and their corresponding consumers are presented. Only attributes checked by over 50% of the total consumers for each product were used for the PA analysis. An attribute not checked by a certain consumer can be checked by another consumer, owing to different subject-specific thresholds. One can easily imagine that consumers will only check an attribute if its intensity exceeds their threshold for the attribute tasted, and they would not check it if it is noticeable but not intense enough. Vidal et al. [31] also stated that 0 on the RATA scale does not necessarily complete the absence of the attribute, whereas it could mean that the intensity is below an individual threshold, and the threshold could differ among individuals.

As shown in Table 4, product A showed a significant proportion of consumers who found sweetness to be too low (75.7%). The calculated penalty (i.e., the mean difference for OL between the group of consumers who found sweetness to be JAR and the group who found it to be too low) was −1.03 on the 9-point hedonic scale, significant both by the t-test and bootstrapping approach. Too strong a “yellowness” in product C indicated that this product would not be acceptable with a significant proportion of consumers (82.2%), with a significant penalty of 1.24. Yang et al. [65] reported that yellow- or orange-colored complexes can be formed due to the different yeast or starter used in rice wine. Low OL for product C (below 4.0, refer to Table 2) may be attributed to this strong “yellowness” However, this is not in agreement with a previous study [64] that reported that such an appearance as “yellowness” showed little or no negative impact on the OL of *yakju*. This inconsistency may be because a larger proportion of consumers (>80%) perceived this product as less sweet in the current study.

Both products D and E showed similar results to those of product A with “sweetness.” A significant proportion of consumers (greater than 50%) found this attribute to be “too little” (penalties 1.31, 1.00, and E, respectively). Strong sweetness has been reported as a key attribute driving liking in *yakju* or rice wine [66,67]. Product F received significant penalties for “ginseng flavor” from both deviations, showing a 1.35 mean drop for the “too little” side (33.6% of consumers) and a 2.28 mean drop for the “too much” side (40.1% of consumers). This indicated that distinct consumer segmentations exist regarding consumers’ perception of the appropriateness of “sweetness” level, or consumers disagreed about what constitutes a suitable level of “sweetness.” Product H showed a significant proportion of consumers who found “mushroom aromatic,” “leaven aromatic,” “leaven flavor,” and “astringency” to be too strong (27.1%–49.5% of consumers). Significant penalties detrimental to OL of this product ranged from 1.03 to 1.25. Referring to Table 2, product H was least liked by consumers, showing an OL of 3.2–3.5.

No texture-related attributes were identified as “too little” or “too much” for the products. Furthermore, texture plays a minor role in dictating consumer satisfaction for most types of liquors, including *yakju* [68].

## 4. Conclusions

The performance of the CATA-JAR, a variant of the CATA method, was examined for the evaluation of Korean traditional rice wine (*yakju*). The sample configurations and discrimination ability obtained from CATA, RATA, and CATA-JAR were compared using RV coefficients. In addition, the frequency of sensory terms, dendrograms generated via AHC analysis, and CA were also investigated for each method. The stability of the sample configurations for each method was determined using a bootstrapping resampling process. Furthermore, for the CATA-JAR method, considerable penalties for each attribute and product were determined using the t-test and bootstrapping technique.

The CATA-JAR method did not cause hedonic bias, suggesting that liking data could be concurrently employed with CATA-JAR questions. The ability of the CATA-JAR method to discriminate samples was better than that of the CATA method, whereas it was better than or similar to the RATA method. The CATA-JAR method also characterized samples and their sensory terms equivalent to the RATA method. Consumers’ analytical cognition elicited from CATA-JAR or RATA may induce somewhat different results from the CATA method. With better performance in discriminating products and stability, CATA-JAR should be implemented if samples with subtle differences in attributes need to be compared. PA, using the combination of CATA questions and JAR scales, allowed the detection of deviations and salient attributes detrimental to the liking of samples. This would be beneficial for product developers or sensory scientists to make decisions regarding the sensory attributes that should be improved to optimize the products. Future research should be extended to research dealing with samples with less heterogeneous sensory attributes for comprehensive performance of the CATA-JAR method.

## Figures and Tables

**Table 1:** Information for the *yakju* used in this study.

Product Code	Alcohol Content (%)	Raw Materials
A	13	Purified water, glutinous rice, glucose, yeast, citric acid, lactic acid, enzyme supplements
B	13	Purified water, ethyl alcohol, high-fructose corn syrup, starch syrup, citric acid, lactic acid, yeast, starter
C	16	Purified water, rice, glutinous rice, popped rice, glucose, isomalto oligosaccharide, platycodon, yeast, starter
D	12.5	Purified water, corn starch, glutinous rice, other fructose, chysanthemum, acacia honey, yeast, purified enzyme, citric acid
E	13	Purified water, rice, starch syrup, yeast
F	12.5	Purified water, rice, ginseng, high-fructose, yeast, starter powder, crude amylolytic enzyme
G	13	Purified water, white rice, yeast, pine bud concentrate
H	14	Purified water, white rice, starter, yeast, citric acid, enzymatically modified stevia glucosyl stevia
I	12.5	Purified water, rice, red ginseng concentrate, high-fructose, yeast, starter powder, crude amylolytic enzyme
J	14	Purified water, glutinous rice, rice, yeast, starter
K	14	Water, rice, yeast
L	11	White rice, yeast, dried orange peel, yeast, crude amylolytic enzyme, purified enzyme, high-fructose corn syrup, citric acid, steviol glycoside

**Table 2:** Overall liking of 12 *yakjus* obtained from the consumers for each of the methods.

	CATA	RATA	CATA-JAR
A	5.1 ^c,d,1)^	5.2 ^c^	5.2 ^b,c^
B	5.0 ^c,d^	5.2 ^c^	4.8 ^c^
C	3.5 ^f^	3.5 ^e,f^	3.9 ^d,e^
D	5.7 ^b,c^	6.0 ^a,b^	5.9 ^a,b^
E	6.5 ^a^	6.2 ^a^	6.3 ^a^
F	5.0 ^c,d^	4.7 ^c,d^	4.4 ^c^
G	3.9 ^e,f^	4.2 ^d,e^	3.9 ^d,e^
H	3.2 ^f^	3.2 ^f^	3.5 ^e^
I	4.5 ^d,e^	4.5 ^c,d^	4.6 ^c,d^
J	5.1 ^c,d^	4.7 ^c,d^	4.8 ^c^
K	5.4 ^b,c^	5.3 ^b,c^	5.3 ^b,c^
L	6.0 ^a,b^	6.0 ^a,b^	6.0 ^a,b^

^1)^ Means not sharing the same superscript letter in the same column are significantly different at *p* < 0.05. No significant differences were observed among the methods.

**Table 3:** Summary of results for the comparison of sensory characterizations with consumers obtained with different methods.

Term Usage		
(a) Percentage of terms used to describe samples	CATA	26.6% ^c,1)^
	RATA	29.4% ^a^
	CATA-JAR	28.7% ^b^
(b) Distribution of intensity scores in RATA method		0: 70.6%
		1: 5.9%
		2: 7.7%
		3: 7.8%
		4: 5.7%
		5: 2.3%
(c) Distribution of JAR scores in CATA-JAR method		0: 71.3%
		1: 4.2%
		2: 6.0%
		3: 10.0%
		4: 6.5%
		5: 2.0%
(d) Percentage of terms with significant differences among samples (*p* < 0.05)	CATA	92.6%
	RATA	100%
	CATA-JAR	100%
**Sample and term configurations**		
(e) Percentage of variance explained by the first two dimensions	CATA	72.2%
	RATA	73.2%
	CATA-JAR	73.1%
(f) RV between sample configurations obtained from CA of data from CATA and RATA method		0.935 *
(g) RV between sample configurations obtained from CA of data from CATA and CATA-JAR method		0.903 *
(h) RV between sample configurations obtained from CA of data from RATA and CATA-JAR method		0.978 *
(i) RV between term configurations obtained from CA data from CATA and RATA method		0.956 *
(j) RV between term configurations obtained from CA data from CATA and CATA-JAR method		0.907 *
(k) RV between term configurations obtained from CA data from RATA and CATA-JAR method		0.958 *

^1)^ Values not sharing the same superscripts in the same column are significantly different at *p* < 0.05. * Indicates that the RV coefficient is significant at *p* < 0.05. CA = Correspondence analysis.

**Table 4:** Summary of penalty analysis obtained from the CATA-JAR method.

Sample/Attribute	% of Consumers	Penalty ^3)^	*p* Value (*t*-Test)	*p* Value (Bootstrapping)
A ^1)^_Alcohol (A) ^2)^	20.0	1.40	0.0010	n.s.
A_Sweetness	75.7	−1.03	0.0072	0.0018
A_Alcohol (F)	20.0	1.03	0.0158	n.s.
C_Yellowness	82.2	1.24	0.0075	0.0021
D_Sweetness	57.0	−1.31	0.0001	0.0000
E_Sweetness	53.2	−1.00	0.0063	0.0039
E_Fruit (F)	48.6	−1.18	0.0008	0.0002
F_Ginseng (A)	58.9	0.83	n.s.	n.s.
F_Ginseng (F)	33.6	−1.35	0.0015	0.0006
F_Ginseng (F)	40.1	2.28	0.0000	0.0000
G_Bitterness	24.3	1.13	0.0278	0.0456
G_Astringency	20.0	0.94	n.s.	n.s.
H_Mushroom (A)	27.1	1.03	0.029	n.s.
H_Leaven (A)	49.5	1.16	0.0089	n.s.
H_Leaven (F)	36.4	1.25	0.0009	n.s.
H_Astringency	30.8	1.06	0.0322	0.0497
I_Yellowness	86.9	0.94	n.s.	n.s.
I_Mushroom (A)	27.1	0.87	n.s.	n.s.
I_Leaven (A)	39.3	1.00	n.s.	n.s.
I_Sweetness	60.7	−1.45	0.0003	0.0001
I_Leaven (F)	27.1	1.56	0.0035	0.0040
K_Sweetness	42.9	−1.62	0.0016	0.0014
K_Malty (F)	48.6	−1.23	0.0055	0.0073
K_Malty (F)	25.2	1.58	0.0026	0.0017
L_Sweetness	53.3	−1.00	0.0034	0.0029
L_Fruit (F)	62.6	−1.07	0.0012	0.0023

^1)^ Product codes as in Table 1. ^2)^ A = Aromatic; F = Flavor. ^3)^ Penalty: (+) too much, (−) too little. n.s = not significant (α = 0.05).
